# Editorial: Climate change and developmental physiology

**DOI:** 10.3389/fphys.2024.1408809

**Published:** 2024-04-25

**Authors:** Sukanta Mondal, Rafael Martinez-Garcia

**Affiliations:** ^1^ Animal Physiology Division, ICAR-National Institute of Animal Nutrition and Physiology, Bangalore, India; ^2^ Universidad Juárez Autónoma de Tabasco, Villahermosa, Mexico

**Keywords:** climate change, developmental physiology, greenhouse gases, fertility, preterm birth, endometrial function, oocyte maturation, developmental potential

Climate change poses the most significant threat to mammalian species due to its potential impacts on heat stress, food and water security, extreme weather events, vulnerability of habitats, and population migration. According to projections by the United Nations (U.N.), the global population is expected to surpass nine billion by 2030, underscoring the urgent need to enhance both the quality and quantity of food production. The U.N. Food and Agriculture Organization (FAO) has emphasized that 70 percent of the required additional food must be sourced from the adoption of novel and existing agricultural technologies. The primary culprit for global climate change is said to be the emission of greenhouse gases (GHG), which results in the warming of the atmosphere (IPCC et al., 2014). Depending on the scale of future GHG emissions, long-term forecasts project an additional rise in global mean temperatures ranging from 1.1°C to 4.8°C above the increases observed over the past 5 decades (IPCC Intergovernmental Panel on Climate Change et al., 2013). Greenhouse gas (GHG) emissions from human activity and livestock are a significant driver of climate change, trapping heat in the Earth’s atmosphere and triggering “Global warming,” which is the ongoing rise of the average temperature of the Earth’s climate. The repercussions of climate change are profound, encompassing elevated atmospheric temperatures, shifts in rainfall patterns, diminished water resources, alterations in the availability and quality of agricultural inputs, and the onset of unprecedented extreme weather events such as elevated sea surface temperatures, sea level rise, and the melting of polar glaciers and ice caps (Stocker et al., 2013).

As we move into the 21st century, the environment faces considerable pressures like forecasting, an increase in the world population to more than 8.5 billion, limitations of available land and water resources, low increases in crop yields, demands for cheaper and safer food and the need to sustain and improve the surrounding environment. Human activities pose ongoing threats, with climate change emerging as a major concern due to its potential to cause profound and possibly irreversible alterations to geological, biological, environmental, and ecological systems. These changes lead to large-scale environmental hazards affecting both living and non-living components, including human health. They include phenomena like extreme weather events, ozone depletion, biodiversity loss, and stresses on food and energy production systems. Nowadays, climate change studies provide knowledge about changing climatic conditions, including heat waves, rapid changes in climatic conditions, severe storms, rising sea levels, and longer and more intense droughts. These investigations have generated different impact scenarios on environmental variables that are having various effects on the environment. The new approaches in this area of study mark a very important line of research, which is how these changes affect or will affect the physiological processes of the various organisms on our planet, because these changes are a direct threat to the organisms, placing them under changing environmental conditions that are sometimes outside their maximum tolerance limits. Organisms, faced with these conditions, must face these extreme and changing conditions, which is why they invest greater energy to maintain homeostasis through changes in their physiology, morphology, and behavior. These changes can generate direct effects on their life cycle, growth, reproduction, and death. It is necessary to undertake more studies that can predict different scenarios of change and adaptation not only in adult organisms but also in developing organisms. This Research Topic arises from the need to generate knowledge in the proposed scenario; information on the effects of climate change on the physiology of developing organisms is scarce. This knowledge is of utmost importance and has scope in our society in various ways.

Elevated environmental temperatures represent a significant factor contributing to decreased fertility (Ealy et al., 1995). Various factors, including suboptimal environmental conditions, management practices, age, and species-specific sensitivities, can depress reproductive performance (Badinga et al., 1985). Particularly, high ambient temperatures alone (Baumgartner and Chrisman, 1981), or combined with humidity, significantly impact reproductive performance in domestic animals during hot seasons. Heat stress disrupts reproduction by causing animals to fail to cope with elevated temperatures, resulting in a rise in body temperature beyond the regulated set point. This can compromise germ cell function and the viability of early embryos (Hansen, 2009). Heat stress leads to considerable suppression in endometrial function (Malayer et al., 1988), compromised follicular growth (Badinga et al., 1985), altered hormonal secretion (Wolfenson et al., 1995; Mondal et al., 2004; Mondal et al., 2007; Mondal et al., 2015a; Mondal et al., 2017a; Mondal et al., 2017b), impaired oocyte maturation (Mondal et al., 2015b; Mondal et al., 2024), and reduced developmental potential (Al-Katanani et al., 2002; Mondal, 2014).

In the Research Topic Climate Change and Developmental Physiology, developmental consequences of short-term and long-term climate change focusing on the effect on the early development of the California purple sea urchin (Leach and Hofmann), short-term exposure to various extreme temperatures on growth, development, reproductive capacity and pest control ability of E. formosa during different developmental stages (Li et al.), association between ambient temperature, humidity, and preterm birth risk, as well as the effects of extreme weather events during early pregnancy and before parturition on preterm birth (Wu et al.) “Bet hedging” against climate change in developing and adult animals: roles for stochastic gene expression, phenotypic plasticity, epigenetic inheritance and adaptation (Burggren and Mendez-Sanchez) are addressed. Additionally, the role of stochastic gene expression, phenotypic plasticity, epigenetic inheritance, and adaptation in “bet hedging” against climate change in developing and adult animals is explored (Burggren and Mendez-Sanchez).

In the investigation by Leach and Hofmann (2024) the authors examined the impact of temperatures akin to Marine Heatwaves (MHWs) on the early development of the California purple sea urchin, *S. purpuratus*, within the framework of paternal thermal history. Their findings indicated that *Strongylocentrotus purpuratus* larvae exhibited the highest thermal tolerance when their development occurred at a temperature matching the paternal acclimation. Interestingly, they observed that larval size remained unaffected by paternal thermal history but was predominantly influenced by the temperature of larval rearing, with higher temperatures yielding significantly larger offspring.

Elevated summer temperatures, greenhouse conditions, extreme winter temperatures, and low-temperature storage techniques during commercial production have been identified to impact the survival and reproductive capabilities of E. formosa, thereby altering its efficacy in controlling Bemisia tabaci. Investigating the effects of short-term exposure to diverse extreme temperatures on the growth, development, reproductive capacity, and pest control capabilities of E. formosa across its various developmental stages, Li et al. aims to provide insights for strategic releases of E. formosa in future agricultural practices. Additionally, it serves as a valuable reference for understanding and predicting E. formosa’s control efficiency in environments characterized by extreme temperatures. The tolerance of E. formosa to temperature extremes varies depending on its developmental stage. Notably, the pupal stage of E. formosa exhibited a high degree of tolerance to both heat and cold, whereas the adult stage displayed weaker tolerance. Exposure to short-term temperature extremes had adverse effects on parameters such as eclosion rate, total parasitism, eclosion rate of the F1 generation, and adult longevity of the F1 generation in E. formosa. Interestingly, the emergence of male individuals in the F1 generation was observed exclusively after exposing the pupal stage of E. formosa to HLT50 treatment.

Preterm birth represents a significant global public health concern, with complications ranking as the second leading cause of death among children under 5 years old worldwide, following pneumonia (Liu et al., 2016). Annually, approximately 944,000 neonates succumb to preterm birth-related complications, constituting 35.3% of all neonatal deaths (WHO, 2018). Notably, in low-income countries, the prevalence of preterm birth averages 12%, while in high-income countries, it stands at 9%. China, with its sizable population and high birth rate, ranks second globally in terms of preterm births (WHO, 2018). Addressing the issue of preterm birth is imperative for reducing neonatal and childhood mortality and morbidity. In their research, Wu et al. conducted research to delve into the effects of environmental factors during pregnancy on preterm birth. They carried out a population-based cohort study involving women aged 18–49 years who participated in the National Free Preconception Health Examination Project (NFPHEP) in Yunnan Province from 1 January 2010, to 31 December 2018. Their study assessed the association between ambient temperature and humidity with preterm birth, as well as the impacts of extreme weather conditions during early pregnancy and just before delivery. Their findings revealed that extremely low temperatures (P1) and low temperatures (P5) at both 1 week and 4 weeks of pregnancy were identified as risk factors for preterm birth. Conversely, extremely low temperatures (P1) and low temperatures (P5) at 4 weeks before delivery and 1 week before delivery acted as protective factors against preterm birth. High temperatures (P95) and extremely high temperatures (P99) were recognized as risk factors for preterm labor across the four exposure windows, with the most pronounced effects observed at 4 weeks before delivery.

Comprehending the impacts of short-term environmental fluctuations on developing animals will be crucial in uncovering the complete effects of global climate change and determining the extent to which organisms can withstand current and future environmental stressors. The review explored the concept of ‘bet hedging’ in fluctuating environments, suggesting that various phenotypes within a population may possess different levels of fitness in any given environment. While this may lower overall population fitness, it ensures the existence of individuals favorably adapted to altered conditions. There are several mechanisms through which phenotypic changes can occur in individuals and populations, including stochastic gene expression, phenotypic plasticity, transgenerational inheritance, and adaptation. These mechanisms, combined with bet hedging, confer significant potential for surviving weather events associated with climate change.

The current Research Topic on Climate Change and Developmental Physiology aims to illuminate the developmental physiological alterations induced by climate change across various animal species, potentially extending to humans. Overall, further elucidation is needed to deepen our understanding of the underlying mechanisms by which climate change impacts developmental processes.
